# Consensus Report of Group 1 of the 1st Global Consensus for Clinical Guidelines for the Rehabilitation of the Edentulous Maxilla: Number of Implants, Timing of Implant Placement and Loading

**DOI:** 10.1111/clr.70063

**Published:** 2026-02-24

**Authors:** Nikos Donos, Ethan Ng, Claudio Mendes Pannuti, Giuseppe Alexandre Romito, Helena Cristina Oliveira Francisco, Samir Abou‐Ayash, Gustavo Avila‐Ortiz, Joao Manuel Mendez Carames, Paolo Casentini, Tali Chackartchi, Vivianne Chappuis, Stephen Chen, Paul Fugazzotto, William V. Giannobile, Yoshiyuki Hagiwara, Adam Hamilton, Saso Ivanovski, Sergio Kahn, Joseph Kan, France Lambert, Robert Alan Levine, Jose Manuel Navarro, Turker Ornekol, Michael Payer, Todd Schoenbaum, Manish Kumar Singh, Sejal Thacker, Gil Alcoforado

**Affiliations:** ^1^ Centre for Oral Clinical Research, Institute of Dentistry, Faculty of Medicine and Dentistry Queen Mary University of London London UK; ^2^ Department of Restorative Dentistry National Dental Centre Singapore Singapore; ^3^ Department of Periodontics Universidade de São Paulo—USP, School of Dentistry São Paulo São Paulo Brazil; ^4^ Instituto de Implantologia Lisbon Portugal; ^5^ Department of Oral Surgery and Oral Medicine Faculdade de Medicina Dentária, Universidade de Lisboa Lisbon Portugal; ^6^ Department of Reconstructive Dentistry and Gerodontology, School of Dental Medicine University of Bern Bern Switzerland; ^7^ Department of Prosthetic Dentistry and Material Science University Medical Center of the Johannes Gutenberg University Mainz Mainz Germany; ^8^ Department of Periodontics and Oral Medicine University of Michigan School of Dentistry Ann Arbor Michigan USA; ^9^ Private Practice Milan Italy; ^10^ Department of Periodontology, Hadassah Medical Center, Faculty of Dental Medicine Hebrew University of Jerusalem Jerusalem Israel; ^11^ Department of Oral Surgery and Stomatology, School of Dental Medicine University of Bern Bern Switzerland; ^12^ Melbourne Dental School The University of Melbourne Parkville Victoria Australia; ^13^ Private Practice Massachusetts USA; ^14^ Department of Oral Medicine, Infection, and Immunity, School of Dental Medicine Harvard University Boston Massachusetts USA; ^15^ Department of Partial Denture Prosthodontics Nihon University School of Dentistry Tokyo Japan; ^16^ Department of Restorative Dentistry and Biomaterials Sciences Harvard School of Dental Medicine Boston Massachusetts USA; ^17^ University of Western Australia Dental School Nedlands Western Australia Australia; ^18^ The University of Queensland Center for Orofacial Regeneration, Rehabilitation and Reconstruction (COR3) Brisbane Queensland Australia; ^19^ School of Dentistry The University of Queensland Brisbane Queensland Australia; ^20^ São Leopoldo Mandic Rio de Janeiro Rio de Janeiro Brazil; ^21^ Department of Implant Dentistry, School of Dentistry Loma Linda University Loma Linda California USA; ^22^ Dental Biomaterial Research Unit University of Liège Liège Belgium; ^23^ Department of Periodontology and Oro‐Dental and Implant Surgery, CHU of Liège University of Liege Liège Belgium; ^24^ Periodontology and Implantology, Kornberg School of Dentistry Temple University Philadelphia Pennsylvania USA; ^25^ Private Practice, Pennsylvania Center for Dental Implants and Periodontics Philadelphia Pennsylvania USA; ^26^ Department of Periodontology and Implant Dentistry New York University College of Dentistry New York New York USA; ^27^ Cosmodent Center for Dentistry and Dental Implants Istanbul Turkey; ^28^ Department of Oral Surgery and Orthodontics University Clinic of Dental Medicine & Oral Health, Medical University of Graz Graz Austria; ^29^ Department of Restorative Sciences Dental College of Georgia, Augusta University Augusta Georgia USA; ^30^ Private Practice Bangalore India; ^31^ Division of Periodontology, UConn Health University of Connecticut School of Dental Medicine Farmington Connecticut USA; ^32^ Egas Moniz School of Health & Science Egas Moniz Center for Interdisciplinary Research Almada Portugal

**Keywords:** consensus conference, edentulous jaw, full‐arch restoration, implant overdenture, implant‐supported dental prosthesis

## Abstract

**Objectives:**

The 1st Global Consensus for Clinical Guidelines (GCCG) in Implant Dentistry introduced an innovative, evidence‐based approach to developing patient‐centered and practical recommendations for the rehabilitation of the edentulous maxilla. Within this framework, Group 1 aimed to formulate clinical recommendations on the number of implants required, timing of implant placement, and timing of loading.

**Materials and Methods:**

Group 1 followed the S2k‐level guideline framework of the Association of the Scientific Medical Societies in Germany (AWMF), using a structured nominal group technique. The evidence base included three systematic reviews evaluating clinician‐reported outcomes (ClinROs) and patient‐reported outcomes (PROs), supplemented by structured single‐round international surveys involving expert clinicians, patients, and cross‐disciplinary experts. Survey content covered diagnostics, treatment planning, clinical procedures, and maintenance care. Draft recommendations were discussed during the in‐person consensus meeting in Boston (June 16–18, 2025) and finalized through anonymous plenary voting. Consensus was defined as ≥ 75% and ≤ 95% agreement and strong consensus as > 95% agreement.

**Results:**

Group 1 formulated 12 clinical recommendations across the workflow domains of diagnostic tools, treatment planning, and treatment procedure. During plenary voting, three of these recommendations reached strong consensus, and nine achieved consensus. The number of voters per recommendation ranged from 61 to 90, with an average of 83.

**Conclusions:**

This consensus report provides structured, evidence‐based recommendations on implant number, placement timing, and loading protocols for rehabilitation of the edentulous maxilla. These guidelines are intended to support individualized, patient‐centered care while also identifying priority areas for future research.

## 1 Introduction

The 1st Global Consensus for Clinical Guidelines (GCCG) in Implant Dentistry introduced an innovative, evidence‐based approach to consensus‐building in implant dentistry. The initiative focused on the rehabilitation of the edentulous maxilla, with the goal of developing patient‐centered and practical clinical recommendations. Working Group 1 specifically addressed key considerations related to the number of implants required, as well as the timing of implant placement and loading.

The recommendations outlined in this report were made by the participants of the 1st GCCG for rehabilitation of patients with edentulous maxillae. It is recognized that two distinct case types may present to dentists for reconstructive therapy—patients with preexisting maxillary edentulism and those with remaining severely compromised maxillary teeth, where treating and maintaining the remaining maxillary dentition is irrational based on the restorative and/or periodontal and/or endodontic status of the teeth.

It is also recognized, and should be stated and emphasized, that the decision to edentulate a patient's maxilla is not one to be made lightly. The preservation of natural dentition and promotion of oral health must always remain the foremost priority. From both an ethical and professional standpoint, every reasonable effort must be undertaken to retain teeth before considering extraction when it is in the best interest of the patient. Any recommendations for prosthetic rehabilitation—whether with or without implants—must be predicated on a thorough evaluation and the conscientious fulfillment of our professional duty to explore all viable options for tooth preservation.

The dentist should engage with the patient in a dialectic manner, carefully considering the patient's concerns and desires, explaining the advantages and risks inherent in each treatment option, and working with the patient to determine the most appropriate therapeutic path for the patient as an individual. For patients considering extraction of their remaining teeth, the discussion must include the potential benefits and risks of retaining the teeth. The care must be delivered with thoughtful consideration of the patient's individual preferences, systemic health, local anatomical conditions, and the practical resources necessary to ensure safe, appropriate, and successful treatment. Rehabilitation of the edentulous maxilla via implant therapy should be performed by clinicians with the requisite expertise, training, and clinical judgment.

For the purposes of implant therapy, an edentulous maxilla is defined as a maxillary arch with complete loss of all natural teeth, regardless of the time elapsed since extractions or the healing stage of the alveolar ridge. In patients considered for extraction of the maxillary dentition and/or rehabilitation of the edentulous maxilla, we recommend that these procedures should be undertaken after careful, comprehensive consideration of all clinical and patient‐specific factors. The definitions used by this working group can be found in Table 1.

**TABLE 1:** Terms and definitions in the consensus report of Group 1.

Terms	Definitions
Implant‐supported prosthesis	Dental prosthesis, such as artificial crown, fixed complete denture, fixed partial denture, removable complete overdenture, removable partial overdenture, as well as maxillofacial prothesis, which are supported and retained in part or whole by dental implants.
Immediate implant placement	Dental implants are placed in the socket on the same day as tooth extraction.
Early implant placement	Dental implants are placed with soft tissue healing (4–8 weeks) or with partial bone healing (12–16 weeks) after tooth extraction.
Late implant placement	Dental implants are placed after complete bone healing, more than 6 months after tooth extraction.
Immediate loading	Dental implants are connected to a prosthesis in occlusion with the opposing arch within 1 week subsequent to implant placement.
Immediate restoration	Dental implants are connected to a prosthesis held out of occlusion with the opposing arch within 1 week subsequent to implant placement.
Early loading	Dental implants are connected to the prosthesis between 1 week and 2 months after implant placement.
Conventional loading	Dental implants are allowed a healing period of more than 2 months after implant placement with no connection of the prosthesis.
FP1	Fixed full‐arch implant restorations that replaces missing teeth with natural looking crowns, without any visible pink restorative material.
FP2	Full‐arch implant restorations that replaces missing teeth with longer crowns without any pink restorative material.
FP3	Fixed full‐arch implant prosthesis that replaces missing hard and soft tissues with natural looking crowns and pink restorative material.

## 2 Methodological Framework and Consensus Procedures of the 1st GCCG

**FIGURE 1:**
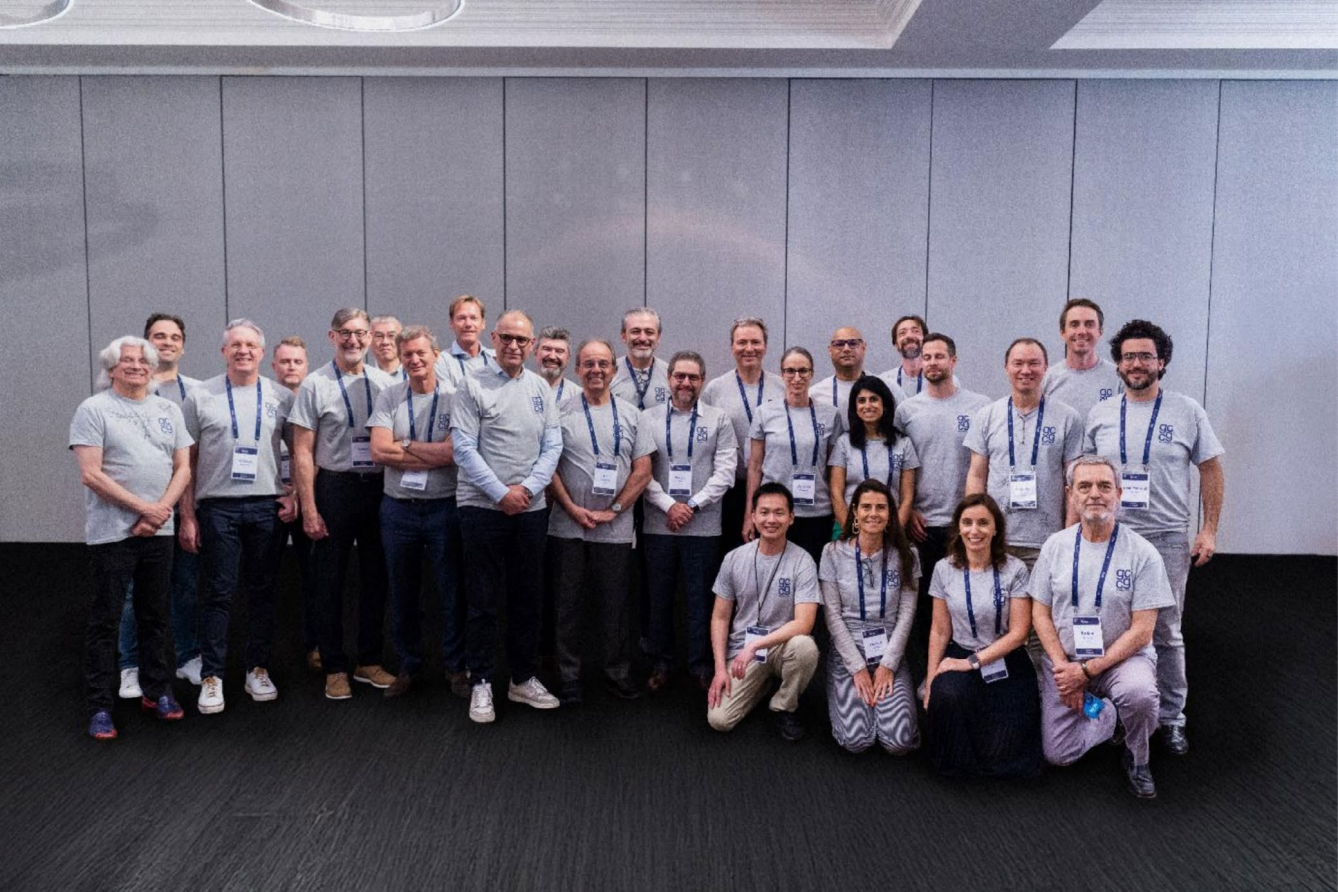
Photograph of participants of Group 1 of the 1st Global Consensus for Clinical Guidelines (GCCG) for the rehabilitation of the edentulous maxilla, gathered during the in‐person consensus conference (Boston, June 2025).

This working group was one of four formed within the 1st Global Consensus Conference for Clinical Guidelines (GCCG) for the “Rehabilitation of the Edentulous Maxilla”, following the S2k‐level framework of the Association of the Scientific Medical Societies in Germany (AWMF). Group 1 comprised 28 of the 105 experts participating in the consensus conference (Figure 1), representing the specialties of prosthodontics (5), periodontics (16), and oral surgery (6), and one general dentist.

Group recommendations were developed through a structured multiphase process combining systematic evidence synthesis with expert and stakeholder input. Preparatory work included systematic reviews on patient‐reported outcome measures (PROMs) and clinician‐reported outcomes (ClinROs) (Francisco et al. 2026; Panutti et al. 2026; Romito et al. 2026), as well as structured surveys involving clinicians, patients, and other stakeholders (Lin et al. 2026; Schoenbaum et al. 2026). These data formed the basis for working group discussions held during the on‐site meeting in Boston (June 16–18, 2025).

Using the Nominal Group Technique, each recommendation was developed and refined through individual review, prioritization voting, and moderated discussion. Recommendations were primarily derived from the results of the structured surveys and reviewed in the context of the available evidence. Where alignment was observed, recommendations were categorized as “aligned with current evidence.” In areas where relevant evidence was lacking, they were classified as “could not be evaluated due to a lack of existing evidence.” Where survey results diverged from available data, recommendations were designated as “not aligned with current evidence.”

The ClinROs and PROMs identified through the systematic reviews and structured surveys served as the foundation for defining a core outcome set (COS), which is a recommended set of outcomes to be reported on in clinical trials and related research for the rehabilitation of the edentulous maxilla. This set was further refined and adopted during the consensus conference using a structured three‐round Delphi process (Schwarz et al. 2026) and served for the formulation of the final recommendations.

Final consensus was reached via anonymous plenary voting, with predefined consensus thresholds (> 95% agreement strong consensus; ≥ 75% and ≤ 95% agreement consensus; < 75% agreement no consensus). Conflicts of interest were disclosed per the International Committee of Medical Journal Editors (ICMJE) standards, and abstentions were documented. The full methodological framework is detailed in the umbrella paper (Schwarz et al. 2026).

## 3 Summary of the Reported Clinician‐ and Patient‐Reported Outcomes

In preparation for the 1st GCCG in Implant Dentistry, eight systematic reviews aimed at identifying and evaluating available evidence regarding clinician‐ and patient‐reported outcomes (ClinROs and PROs) and their corresponding assessment measures were conducted. This section summarizes the most relevant findings from the three systematic reviews (Francisco et al. 2026; Panutti et al. 2026; Romito et al. 2026) that informed the discussions of Working Group 1, with regard to ClinROs and PROs used in studies that evaluated the number of implants used, timing of implant placement, and implant loading in the rehabilitation of the edentulous maxilla. Studies involving implants placed in the pterygoid or zygomatic bones were excluded.

### 3.1 ClinROs

Clinician‐reported outcomes (ClinROs) and clinician‐reported outcome measures (CROMs) were identified through structured data extraction from the primary studies included in three systematic reviews (Francisco et al. 2026; Panutti et al. 2026; Romito et al. 2026). The ClinROs summarized below represent those most frequently reported in studies evaluating the number of implants, timing of implant placement, and timing of loading in the rehabilitation of the edentulous maxilla.

#### 3.1.1 Implant survival

Survival was defined as the presence of a functional implant (de Araújo Nobre et al. 2020; Testori et al. 2021; Van Doorne et al. 2020) without signs of mobility (Bouhy et al. 2023; Boven et al. 2017). Implant survival was a composite outcome which was assessed dichotomously (yes/no).

#### 3.1.2 Implant success

The composite implant success criteria were determined by no clinical detectable implant mobility; absence of neurological symptoms or problems; no recurrent peri‐implant infection and no continuous radiolucency around the implant (Zhang et al. 2016). This was assessed dichotomously (yes/no).

#### 3.1.3 Peri‐implant marginal bone loss

Peri‐implant marginal bone loss was measured in millimeters, typically from the implant platform or collar to the most coronal bone‐to‐implant contact at mesial and distal sites using standardized periapical radiographs (Boven et al. 2017; Tallarico et al. 2016; Yamada et al. 2015; Zhang et al. 2016).

#### 3.1.4 Peri‐implant tissue parameters

Probing depth was assessed using a periodontal probe at multiple sites around each implant, typically applying a force of 20–25 g with a periodontal probe, and reported in millimeters. Measurements were taken at four sites per implant (Bouhy et al. 2023; Boven et al. 2017) or at six sites (Zhang et al. 2016).

Bleeding on probing (BOP) was assessed using a 0.5‐mm diameter periodontal probe typically applying a force of 20–25 g. The Sulcular Modified Bleeding Index (Mombelli et al. 1987) was the most commonly used index used (de Araújo Nobre et al. 2020; Tallarico et al. 2016; Zhang et al. 2016). BOP was measured dichotomously (yes/no) and recorded at 4–6 sites per implant, expressed as percentage of total bleeding sites.

Suppuration on probing (SOP) assessed using a 0.5‐mm diameter periodontal probe at 20–25 g was measured dichotomously (yes/no) and recorded at 4–6 sites per implant (Zhang et al. 2016).

Presence of plaque (microbial biofilm) was assessed using either the plaque index (Loe and Silness 1963) or the modified plaque index (Mombelli et al. 1987). Plaque index was assessed using an ordinal score (e.g., 0 to 3), and recorded at 4–6 sites per implant (Bouhy et al. 2023; Boven et al. 2017; de Araújo Nobre et al. 2020; Tallarico et al. 2016; Zhang et al. 2016).

#### 3.1.5 Biological complications

The following definitions are based on the World Workshop Classification of Periodontal and Peri‐implant diseases and Conditions, jointly presented by the American Academy of Periodontology (AAP) and the European Federation of Periodontology (EFP).

Peri‐implant mucositis was defined as the presence of bleeding and/or suppuration on gentle probing, with or without increased probing depth compared to previous examinations, and no bone loss beyond initial remodeling (Renvert et al. 2018). Peri‐implant mucositis was a composite outcome which was assessed dichotomously (yes/no).

Peri‐implantitis was defined as the presence of bleeding and/or suppuration on probing, increased probing depth compared to previous examinations, and presence of bone loss beyond crestal bone level changes from initial bone remodeling (Renvert et al. 2018). In the absence of previous examination data, bone loss ≥ 3 mm combined with BOP and/or suppuration. Peri‐implantitis was a composite outcome which was assessed dichotomously (yes/no).

#### 3.1.6 Prosthesis survival

Prosthesis survival was defined as the presence of a prosthesis in function (Testori et al. 2021; Van Doorne et al. 2020), with failure defined as the need for prosthesis replacement (de Araújo Nobre et al. 2020; Tallarico et al. 2016; Yamada et al. 2015). Prosthesis survival was assessed dichotomously (yes/no).

#### 3.1.7 Prosthesis success

A prosthesis was regarded as successful if it remained unchanged and no intervention was necessary during the time of observation (Zhang et al. 2016); or as no evidence of retreatment beyond accepted prosthodontic maintenance events (Bouhy et al. 2023). Prosthesis success was assessed dichotomously (yes/no).

#### 3.1.8 Prosthetic complications

Prosthetic complications were defined as the occurrence of mechanical or technical issues involving the prosthesis over a given period of time. These were assessed dichotomously (absent/present) and included events such as fixation screw loosening or fracture, framework or veneering material fracture, and loss of retention. Provisional complications, including misfit of the provisional prosthesis and major occlusal adjustments were also reported (Yamada et al. 2015). In studies evaluating overdentures, complications included assessment of matrix unscrewing, fracture, loss or replacement due to significant wear; matrix fracture or replacement; dislocation, loss and wear of the matrix component (female nylon inserts); overdentures maintenance such as fracture, puncture, reline or prosthesis modification necessary to reposition a new matrix after replacement of a failing implant (Bouhy et al. 2023).

#### 3.1.9 Esthetic satisfaction

Visual analogue scales (VAS) were used to assess clinician satisfaction on esthetics. This was reported as VAS for specific esthetic domains, including: abutment visibility, papilla appearance and presence, esthetic characteristics of restorations, presence of pink ceramics, and external patient appearance (Marković et al. 2022).

#### 3.1.10 Speech

Articulation was evaluated using a structured phonetic examination (Fonteyne et al. 2019). The evaluation used a picture naming test with 135 images of common subjects and actions, covering all Dutch phonems. Speech samples were digitally recorded with a video camera. Phonetic inventory and analysis were conducted, with a sound included if produced at least twice. Two speech‐language therapists independently rated the samples.

#### 3.1.11 Function

Masticatory function was evaluated via occlusal force assessment and electromyographic (EMG) activity (Montero et al. 2021).

#### 3.1.12 Other reported outcomes

Surgery procedure duration: The time elapsed from beginning to end of surgical intervention (Vercruyssen et al. 2016; Yamada et al. 2015).

Implant stability: Assessed using resonance frequency analysis (RFA) (Marković et al. 2022).

Implant insertion torque: Measured in Ncm (Marković et al. 2022; Yamada et al. 2015).

Postoperative swelling: Assessed using a scale of 0 to 4 (Yamada et al. 2015).

Pain, swelling, mobility (Tallarico et al. 2016)

Soft tissue pathology for example, abscess, fistulae (de Araújo Nobre et al. 2020).

A summary of the clinician‐reported outcomes (ClinROs) and their corresponding assessment measures (CROMs), as identified in the systematic reviews and described above, is presented in Table 2.

**TABLE 2:** Summary of ClinROs and CROMs.

ClinROs	CROMs (specific measure) and definition	Relevance to subtopic
Implant survival	Implant survival (dichotomous, composite outcome): implant present and functional, without signs of mobility.	Number of implants; Timing of implant placement
Implant success	Implant Success (dichotomous, composite outcome): absence of pain, infection, mobility, radiolucency.	Number of implants; Timing of implant placement; Timing of implant loading
Peri‐implant marginal bone loss	Peri‐implant marginal bone loss (continuous outcome): radiographic measurement in mm from implant collar to most coronal bone‐to‐implant contact.	Number of implants; Timing of implant placement; Timing of implant loading
Probing depth	Probing depth (continuous outcome): measured with a probe, in mm, at 4–6 sites per implant.	Number of implants; Timing of implant placement; Timing of implant loading
Bleeding on probing (BOP)	Sulcular Modified Bleeding Index: recorded at 4–6 sites per implant, expressed as % of bleeding sites.	Number of implants; Timing of implant placement; Timing of implant loading
Suppuration on probing	Suppuration on probing (dichotomous outcome): recorded at 4–6 sites per implant (yes/no).	Number of implants; Timing of implant placement; Timing of implant loading
Plaque	Plaque Index (Loe and Silness 1963) (ordinal outcome) or modified Plaque Index (Mombelli et al. 1987) (ordinal outcome)	Number of implants; Timing of implant placement; Timing of implant loading
Peri‐implant mucositis	Peri‐implant mucositis (dichotomous, composite outcome): Presence of bleeding and/or suppuration on gentle probing, with or without increased probing depth compared to previous examinations. Peri‐implant mucositis was a composite outcome which was assessed dichotomously (yes/no).	Number of implants; Timing of implant placement; Timing of implant loading
Peri‐implantitis	Peri‐implantitis (dichotomous, composite outcome): Presence of bleeding and/or suppuration on probing, increased probing depth compared to previous examinations, and presence of bone loss beyond crestal bone level changes from initial bone remodeling. In the absence of previous examination data, bone loss ≥ 3 mm combined with BOP and/or suppuration.	Number of implants; Timing of implant placement; Timing of implant loading
Prosthesis survival	Prosthesis survival (dichotomous outcome): prosthesis in function. Failure defined as need for replacement.	Number of implants; Timing of implant placement; Timing of implant loading
Prosthesis success	Prosthesis success (dichotomous, composite outcome): prosthesis remained unchanged, with no retreatment beyond prosthodontic maintenance events.	Number of implants; Timing of implant placement; Timing of implant loading
Prosthetic complications	Prosthetic complications (dichotomous outcome): mechanical or technical events such as screw loosening, fracture, or prosthesis misfit.	Number of implants; Timing of implant placement; Timing of implant loading
Esthetics	Clinician esthetic satisfaction. Reported as visual analog scale (VAS) for six esthetics domains	Timing of implant placement; Timing of implant loading
Speech	Articulation: assessed using a picture naming test with 135 images to elicit all Dutch phonemes. Speech samples were video‐recorded and rated by two independent speech‐language experts.	Number of implants; Timing of implant placement
Masticatory function	Occlusal force assessment (continuous) or electromyographic (EMG) activity (continuous).	Number of implants; Timing of implant placement; Timing of implant loading
Biological complications	Biological complications (dichotomous, outcome): pain, swelling, mobility, infection, soft tissue pathology.	Number of implants; Timing of implant placement; Timing of implant loading
Postoperative swelling	Facial Swelling Scale (ordinal outcome)	Number of implants; Timing of implant placement
Surgery duration	Surgery duration (continuous outcome): time from start to end of procedure.	Number of implants; Timing of implant placement
Surgical complexity	Surgical complexity (continuous outcome): clinician‐rated via visual analog scale (VAS).	Number of implants; Timing of implant placement
Implant stability	Resonance Frequency Analysis (RFA) (continuous outcome): measured with Osstell or similar device.	Number of implants; Timing of implant placement; Timing of implant loading
Implant insertion torque	Insertion torque (continuous outcome): recorded in Ncm at implant placement.	Timing of implant placement

### 3.2 PROs/PROMs

The patient‐reported outcomes (PROs) and associated patient‐reported outcome measures (PROMs) summarized below were the most frequently reported in studies evaluating the number of implants, timing of implant placement, and timing of loading in the rehabilitation of the edentulous maxilla (Francisco et al. 2026; Panutti et al. 2026; Romito et al. 2026). In most studies, PROs were measured using numeric VAS, single‐item scales, standardized or custom questionnaires. Additionally, versions of the Oral Health Impact Profile [OHIP] instruments were frequently used to assess Oral Health‐related Quality of Life.

#### 3.2.1 Morbidity

Postoperative pain and discomfort was frequently assessed with a visual analogue scale (VAS), which is a line of 0–10 cm (100 mm) length that has an anchor at each end representing the extremes of the experience measured for example, no pain and the worst possible pain (Fürhauser et al. 2016; Peñarrocha‐Oltra et al. 2014; Pomares‐Puig et al. 2023; Yamada et al. 2015). Some studies used verbal rating scales comprising 6 levels, from “no pain” to “worst possible pain” (Menini et al. 2016), or 1–10 Numeric Rating Scale categories, interpreted as mild (1–3), moderate (4–6), and severe pain (7–10) (Van Doorne et al. 2020). Pain was recorded at multiple postoperative time points or daily during the first postoperative week.

Swelling was evaluated using VAS (0–10) (Fürhauser et al. 2016; Peñarrocha‐Oltra et al. 2014), or using a clinical ordinal scale from 0 (no visible swelling) to 4 (severe facial swelling) (Yamada et al. 2015). Two studies reported swelling descriptively (Menini et al. 2016; Testori et al. 2021).

Pain, bruising, facial swelling, and effect on chewing/sleeping/speaking were evaluated as part of a 10 question postsurgical quality of life assessment using a 0–10 numerical scale (Testori et al. 2021).

#### 3.2.2 Patient satisfaction

Overall patient satisfaction was commonly assessed via VAS (Bouhy et al. 2023; Peñarrocha‐Oltra et al. 2014; Zembic et al. 2019), Likert‐type questions (Montero et al. 2021), or rating scales (Boven et al. 2017). Satisfaction was also assessed verbally (Zhang et al. 2016) or using OHIP‐derived tools (Fonteyne et al. 2019). Some studies analyzed changes over multiple time points (Peñarrocha‐Oltra et al. 2014; Zembic et al. 2019).

Patient comfort was frequently included in visual analogue scales ranging between 0 and 10 (poor to excellent) (Bouhy et al. 2023; de Araújo Nobre et al. 2020). In addition, Bouhy et al. (2023) used an adaptation of the McGill Denture Satisfaction scale (100 mm VAS).

Prosthesis stability was assessed as one of the domains from the McGill Denture Satisfaction scale with a VAS (Bouhy et al. 2023).

Cleansability was assessed using VAS questions specifically related to ease of cleaning (Peñarrocha‐Oltra et al. 2014), or cleaning ability as one of the domains from the McGill Denture Satisfaction scale (Bouhy et al. 2023).

#### 3.2.3 Function

Self‐reported assessments of speech included VAS ratings (Bouhy et al. 2023; Peñarrocha‐Oltra et al. 2014) and a specific item from the OHIP‐14 questionnaire addressing difficulty pronouncing words (Fonteyne et al. 2019).

Masticatory function was assessed through various tools, including VAS scores for chewing ability and verbal ratings of masticatory ability, with patients rating ability as “excellent,” “good”, “fair”, or “poor” (Bouhy et al. 2023; de Araújo Nobre et al. 2020; Peñarrocha‐Oltra et al. 2014). A chewing ability questionnaire focused on eating soft, tough, and hard foods, scored from 0 (good) to 2 (bad) (Boven et al. 2017; Zhang et al. 2016), and another study assessed self‐reported chewing ability (Montero et al. 2021).

#### 3.2.4 Oral health‐related quality of life

OHRQoL was primarily assessed using versions of the Oral Health Impact Profile (OHIP), including OHIP‐14 (Fonteyne et al. 2019; Misumi et al. 2015; Pomares‐Puig et al. 2023), OHIP‐J14 (Misumi et al. 2015), OHIP‐J49 (Yamada et al. 2015), OHIP‐49 (Erkapers et al. 2017), OHIP‐20/OHIP‐EDENT (Bouhy et al. 2023; Zembic et al. 2019), and OHIP‐19 (Marković et al. 2022).

These instruments cover multiple domains including functional limitation, physical pain, psychological discomfort, physical and psychological disability, social disability, and handicap. Scoring systems varied by version but typically used Likert scales ranging from 0 (“never”) to 4 or 5 (“very often”/“always”), with higher scores indicating worse quality of life. Results were commonly reported as mean (SD) per domain and for total OHIP scores.

A summary of the patient‐reported outcomes (PROs) and their respective assessment measures (PROMs), as identified in the systematic reviews and detailed above, is presented in Table 3.

**TABLE 3:** Summary of PROs and PROMs.

PROs	PROMs (specific measure and definition)	Relevance to subtopic
Postoperative Pain and Discomfort	Visual analog scale (VAS) of postoperative pain (continuous outcome): 0–10 or 0–100 mm scale. Verbal Rating Scale (VRS) (ordinal outcome): six‐point verbal rating scale with categories ranging from “no pain,” to “worst possible pain” Numeric Rating Scale (NRS) (ordinal outcome): numeric 1–10 scale, interpreted as mild, moderate and severe pain.	Number of implants; Timing of implant placement; Timing of implant loading
Postoperative Swelling	Visual analog scale (VAS) of patient‐reported swelling (continuous outcome). Level of patient‐reported facial swelling severity (ordinal scale, 0–4)	Number of implants; Timing of implant placement; Timing of implant loading
Bruising	Patient‐rated bruising intensity (0–10 scale).	Number of implants
Overall Satisfaction	Visual analog scale (VAS): general satisfaction (continuous outcome) Likert‐type questions (ordinal outcome) Rating scales: structured satisfaction ratings. Adaptation of the McGill Denture Satisfaction scale, using a 100 mm VAS Satisfaction embedded in quality‐of‐life instruments (one of OHIP items).	Number of implants; Timing of implant placement; Timing of implant loading
Comfort	Visual analogue scale of perceived comfort with prosthesis, ranging 0–10 (poor to excellent) 100 mm VAS, comfort embedded in the adaptation of the McGill Denture Satisfaction Scale	Number of implants; Timing of implant placement; Timing of implant loading
Stability of Prosthesis	100 mm VAS, stability embedded in the adaptation of the McGill Denture Satisfaction Scale	Number of implants; Timing of implant placement; Timing of implant loading
Cleansability	Visual analogue scale of ease of cleaning 100 mm VAS, cleansability embedded in the adaptation of the McGill Denture Satisfaction Scale	Number of implants; Timing of implant placement
Speech	Visual analogue scale of perceived clarity of speech Speech embedded in quality‐of‐life instruments (one of OHIP items: difficulty pronouncing words.)	Number of implants; Timing of implant placement
Mastication	Visual analogue scale of ease of chewing. Verbal ratings of masticatory ability (ordinal) ranging from “excellent,” to “poor.” Chewing ability questionnaires: food‐type‐based scoring.	Number of implants; Timing of implant placement; Timing of implant loading
Oral Health‐related Quality of Life (OHRQoL)	Different versions of the OHIP: OHIP‐49: 49‐item extended profile. OHIP‐14: 14‐item version. OHIP‐J49: Japanese version of OHIP‐49. OHIP‐J14: Japanese version of OHIP‐14. OHIP‐EDENT: OHIP adapted for edentulous individuals. OHIP‐20: shortened version for edentulous patients. OHIP‐19: another validated short version of OHIP.	Number of implants; Timing of implant placement; Timing of implant loading

### 3.3 Key Observations

There was substantial heterogeneity in both PROs and ClinROs across studies, particularly regarding follow‐up durations and the standardization of measurement instruments.

The use of validated tools was limited. For instance, although versions of OHIP survey were commonly employed to assess OHRQoL, several studies relied on nonvalidated questionnaires, hindering meaningful comparison of findings. Furthermore, even when validated instruments were used, they were often not specifically developed or validated to assess outcomes in the rehabilitation of the edentulous maxilla with implant‐supported prostheses.

Reporting on outcome assessor training and calibration procedures was minimal or absent in most studies.

No consistent pattern emerged regarding how specific ClinROs and PROs align with the type of comparison made in clinical studies (e.g., number of implants, timing of implant placement, or timing of implant loading), underscoring the need for greater harmonization and standardization of outcome selection.

### 3.4 Interventions Addressed in the Reviewed Literature

#### 3.4.1 Number of Implants for Full‐Arch Rehabilitation Using a Fixed Prosthesis

When rehabilitating the edentulous maxilla with a full‐arch fixed prosthesis and implants, two rehabilitation concepts are more commonly used. The first is the “All‐on‐4” concept, which involves placing four implants—typically two anterior straight implants and two posterior tilted implants. This approach is designed to avoid anatomical limitations such as the maxillary sinus and aims to reduce the need for additional bone augmentation. Additionally, this approach may offer a shorter treatment time and lower cost by using fewer implants compared to traditional protocols, which include the use of six or more axially placed implants to increase the distribution of occlusal forces and minimize prosthetic cantilever length.

In terms of outcomes, clinical research on the number of recommended implants to be placed has largely focused on ClinROs such as implant survival, prosthesis survival, and prosthetic complications. PROs commonly used in these studies include satisfaction, comfort, chewing, speech, and Oral Health‐related Quality of Life. However, the utilization of outcomes across studies has been inconsistent, and no standardized outcome set has been clearly associated with studies comparing different numbers of implants.

#### 3.4.2 Timing of Implant Placement (Fixed and Removable Prostheses)

Immediate Implant Placement

*Definition:* Implants are placed immediately after tooth extraction.

*Advantages:* Reduced number of surgical procedures, reduced overall treatment time, optimal availability of existing bone, potentially better preservation of soft tissue contours (if a well‐designed provisional is used), and ability to immediately temporize with a fixed restoration for improved patient satisfaction.

*Disadvantages:* Technique sensitive and usually performed with simultaneous bonewe augmentation; however, adjunctive surgical procedures may still be required.

Early Implant Placement

*Definition:* Implants are placed between 4 and 8 weeks after tooth extraction; soft‐tissue healing has progressed but prior to complete bone healing.

*Advantages:* Resolution of inflammatory local pathology, increased soft tissue volume facilitating flap management, and shorter overall treatment time compared to a delayed approach.

*Disadvantages:* Technique sensitive and usually performed with simultaneous bone augmentation, however, adjunctive surgical procedures may still be required.

Delayed Implant Placement

*Definition:* Implant placement procedure is done at least 3–4 months after tooth extraction for complete or near‐complete bone healing (Tonetti et al. 2019). In the definition by the International Team for Implantology (ITI), which was used in this consensus conference, this healing period coincides with early implant placement with partial bone healing (12–16 weeks) (Gallucci et al. 2018). The ITI also refers to late placement as a healing period of >6 months after tooth extraction.

*Advantages:* More predictable bone remodeling, favorable surgical site assessment and placement, and widely established as a “traditional” protocol.

*Disadvantages:* Extended total treatment time and possibly extensive bone resorption (if alveolar ridge preservation is not done) that may require ridge augmentation.

Alignment Between ClinROs/PROs and Studies on Timing of Implant Placement

- Studies on the timing of implant placement often evaluated ClinROs such as insertion torque, surgical duration, and peri‐implant marginal bone levels.
- PROs most frequently used included postoperative pain and swelling, and, less frequently, assessment of quality of life.
- Outcome selection varied considerably across studies, and no consistent association was observed between specific outcomes and studies on the timing of implant placement.

#### 3.4.3 Timing of Implant Loading (Fixed and Removable Prostheses)

Immediate Loading

*Definition:* The prosthesis (temporary or definitive) is connected to implants within 1 week (often the same day) of implant insertion.

*Advantages:* High patient acceptance (eliminates the need to wear a removable provisional prosthesis) and potential preservation and shaping of soft tissues with a suitable provisional prosthesis.

*Requirements:* Favorable clinical situation (e.g., adequate insertion torque or ISQ resonance frequency) (Javed and Romanos 2010).

*Disadvantages:* Limited evidence suggests a slightly higher risk of implant failure compared to early or conventional loading (Chen et al. 2019; Sanz‐Sánchez et al. 2015; Zhang et al. 2017).

Early Loading

*Definition:* Loading between 1 week and 2 months after implant placement. *Advantages:* Reduces time without teeth replacement or the need to wear a removable provisional prosthesis.

Conventional Loading

*Definition:* Prosthesis is delivered at more than two months after implant placement.

*Advantages:* The most well documented approach, predictability.

*Disadvantages:* The patient is edentulous for an extended period or must use a removable provisional prosthesis during healing.

Alignment Between ClinROs/PROs and Studies on Timing of Implant Loading

- Studies on the timing of implant loading commonly evaluated ClinROs such as implant survival, prosthesis success, and/or prosthetic complications.
- The PROs most frequently assessed included function and satisfaction, Oral Health‐related Quality of Life, and mastication ability.
- Reported outcomes varied across studies, and no consistent alignment was identified between outcome selection and studies on the timing of implant loading.

## 4 Summary of the key findings from the surveys

To complement the evidence synthesized from systematic reviews, two single round surveys were conducted. The first involved 202 invited experts from 42 different countries, achieving a 58% response rate (Schoenbaum et al. 2026). The second included 68 invited patients and 68 cross‐disciplinary experts, reaching a response rate of 60% and 31% respectively (Lin et al. 2026). The objective was to identify prevailing practices, preferences, and areas of consensus regarding the diagnosis, planning, execution, and maintenance of implant‐supported rehabilitations in the edentulous maxilla. The findings are summarized below. Full survey results are available at: “Click here”.

### 4.1 Diagnostic Tools

Among experts, there was strong consensus (83.8%) regarding the routine use of cone‐beam computed tomography (CBCT) in the diagnostic phase of full‐arch rehabilitation.

Similarly, both patients (92.7%) and cross‐disciplinary experts (90.5%) expected the use of CBCT for implant treatment planning. Despite this consensus, concerns about radiation exposure were noted by both patients (36.6%) and cross‐disciplinary experts (57.1%).

### 4.2 Treatment Planning

There was a strong consensus (90%) among experts that implant overdentures should be offered as a treatment option to fully edentulous patients. Experts also showed a strong preference for guided surgery (90%) over freehand techniques. With regards to the number of implants, 72.6% favored using six implants for fixed full‐arch restorations, while for maxillary overdentures, 81.2% preferred four implants. The most frequently selected implant positions were the lateral incisors anteriorly (47%) and the first molars posteriorly (80.3%). When there was sufficient interseptal bone at maxillary molar sites, 70% of experts reported performing immediate implant placement, while 10.2% would not. Regarding loading, 76% indicated they proceed with immediate loading when primary stability is achieved.

Furthermore, 66.7% preferred delivering a fixed provisional restoration when loading immediately. Additionally, 71% of experts preferred FP1 prosthesis (tooth‐only design) with minimal alveoloplasty over an FP3 design requiring alveoloplasty.

In terms of patient preferences, a fixed maxillary full‐arch prosthesis was favored over removable prostheses by 83% of patients and 76% of cross‐disciplinary experts. Esthetic outcomes of the prosthesis were rated as highly important by the vast majority of respondents (95% of patients, 86% of cross‐disciplinary experts).

### 4.3 Treatment Procedure

Regarding provisionalization, 76.8% of experts reported delivering the provisional restoration on the same day or the day after surgery. Laboratory‐fabricated acrylic resin (PMMA) was the preferred material for provisional restorations (83.8%), while definitive restorations were most commonly fabricated using monolithic zirconia on titanium bases (53.9%). Occlusal schemes differed based on the opposing arch: bilateral balanced occlusion was preferred when opposing a removable prosthesis (70%), while mutually protective occlusion (47.9%) or bilateral balanced (33.3%) were chosen when opposing a fixed prosthesis. Regarding the timing for making a definitive impression or digital scan after implant placement in a healed maxillary ridge, 47.0% of experts preferred waiting at least 3 months.

From the patient/cross‐disciplinary expert perspective, fixed provisional prostheses were preferred by 58.5% of patients and 47.6% of cross‐disciplinary expert, although a significant proportion reported no preference. Regarding the impression or scan method, 41.5% of the patients and 42.9% of cross‐disciplinary experts preferred a digital scan, while none of the patients and only 4.8% of experts preferred a conventional impression. The remaining respondents in both groups expressed no specific preference.

### 4.4 Maintenance Care

The importance of regular supportive peri‐implant maintenance care was emphasized by both experts and stakeholders. Experts estimated the average time before needing replacement of retention components in an implant overdenture was every 1 to 2 years (53.8%) or every 2 to 5 years (35%).

In terms of the frequency of follow‐up visits during the first year, 57.1% of cross‐disciplinary experts recommended check‐ups every 3 months, 19.0% suggested every 6 months, and 23.9% favored annual visits. Among patients, preferences differed: 19.5% preferred visits every 3 months, 39.0% every 6 months, and 41.5% once a year.

## 5 Group Recommendations

Group recommendations were formulated through a structured, multiphase process that integrated systematic evidence synthesis with expert and stakeholder input. Recommendations were classified as aligned with current evidence, not aligned, or unable to be evaluated due to insufficient data. Final consensus was achieved via anonymous plenary voting using predefined thresholds (> 95% agreement, strong consensus; ≥ 75% and ≤ 95% agreement, consensus; < 75% agreement, no consensus), with conflicts of interest (CoI) disclosed in accordance with ICMJE standards.

Recommendation No. 1 (Aligned with current evidence).

**Table:** 

Domain	Treatment planning
Recommendation	For patients in need of maxillary arch rehabilitation requiring a complete implant‐supported prosthesis, we recommend that both removable implant overdentures and a fixed prosthesis are offered as treatment options for patients to make an informed decision.
Expert survey results	Most experts (90%) always offer implant overdentures or when indicated.
Patient/cross‐disciplinary expert survey results	83% patients and 76% cross‐disciplinary experts preferred a fixed maxillary full‐arch prosthesis over a removable solution.
Supporting/Contradicting	Edentulous patients rated highly both removable and fixed implant‐supported prostheses (Feine et al. 2018). However, they rated their ability to maintain their oral hygiene significantly higher with the removable prosthesis. Patients preferred fixed protheses when compared to removable (Messias et al. 2023). Similar and low cumulative implant loss for either a removable or fixed implant‐supported full‐arch prosthesis (Ramanauskaite et al. 2022). Both implant‐assisted maxillary reconstructions achieved high prosthetic and implant survival rates, but the observed prosthetic success was significantly lower than prosthetic survival regardless of a fixed or implant overdenture prosthesis (Ng et al. Forthcoming).
Lack of literature	N/A
Evidence	Based on three systematic reviews (Messias et al. 2023; Ng et al. Forthcoming; Ramanauskaite et al. 2022) and one consensus report (Feine et al. 2018), both removable and fixed implant‐supported reconstructions were considered viable treatment options for rehabilitating the edentulous maxilla.
Recommended ClinROs	– Implant failure – Implant success – Implant survival – Mechanical/technical complications – Plaque index/oral hygiene – Prosthetic failure – Prosthetic success – Prosthetic complications – Postoperative complications – Radiographic marginal bone loss – Surgical/intra‐operative complications – Biological complications – Peri‐implant health (implant level) – Peri‐implant health (patient level) – Peri‐implant mucositis – Peri‐implant suppuration – Peri‐implantitis – Clinician's treatment success – Prosthodontic maintenance events/complications
Recommended PROs	– Esthetic satisfaction – Chewing function/comfort/discomfort – Complications during treatment/maintenance – Ease of cleaning/oral hygiene efficiency – Patient overall satisfaction with treatment – Patient‐reported complaints – Prosthesis retention/stability – Quality of life (Oral Health‐Related Quality of Life, OHRQoL) – Speech/phonetics/pronunciation/function
Strength of consensus	Agree: 98% (Strong consensus) Agree: 85/Disagree: 2/Abstain: 0/Abstain (CoI): 0

Recommendation No. 2 (Aligned with current evidence).

**Table:** 

Domain	Diagnostic tools
Recommendation	For patients in need of maxillary arch rehabilitation requiring a complete implant‐supported prosthesis, we recommend the use of CBCT scans as a diagnostic tool to improve accuracy of implant placement and reduce the risk of surgical complications.
Supporting expert survey results	For 84% of experts, the use of CBCT has become a routine treatment planning protocol.
Supporting patient/cross‐disciplinary expert survey results	The majority of patients (92.7%) and cross‐disciplinary experts (90.5%) expected the use of CBCT scans for implant planning.
Supporting/Contradicting	The EAO recommended the use of preoperative cross‐sectional imaging for the following scenarios in the maxilla: (i) where extensive bone augmentation or sinus floor elevation procedures are anticipated (ii) for all guided implant surgery (iii) when planning the use of special surgical techniques such as zygomatic implants. However, it was recommended that if the clinical evaluation showed sufficient bone width and conventional radiographs clearly defined anatomical boundaries and adequate bone height and volume, no additional imaging was mandatory for implant placement (Harris et al. 2012). CBCT can be considered an appropriate diagnostic tool for 3D preoperative planning (Fokas et al. 2018).
Lack of literature	There were no direct studies comparing the necessity of CBCT to 2D radiographs for treatment planning.
Evidence	The use of CBCT scans for implant planning was supported by the EAO consensus report (Harris et al. 2012), one systematic review (Fokas et al. 2018), expert survey (Schoenbaum et al. 2026), and patient and cross‐disciplinary expert surveys (Lin et al. 2026).
Recommended ClinROs	– Surgical/intraoperative complications – Clinician's treatment success
Recommended PROs	– Patient overall satisfaction with treatment – Patient‐reported complaints
Strength of consensus	Agree: 97% (Strong consensus) Agree: 83/Disagree: 3/Abstain: 0/Abstain (CoI): 0

Recommendation No. 3 (Aligned with current evidence).

**Table:** 

Domain	Treatment planning/Treatment procedure
Recommendation	For patients in need of maxillary arch rehabilitation requiring a complete implant‐supported prosthesis, we recommend a prosthetically guided approach as opposed to completely freehand to improve accuracy of implant placement, reduce the risk of surgical complications, and facilitate immediate‐loading procedures.
Supporting expert survey results	The majority of experts (90%) prefer guided surgery approaches over freehand. Among these, 60% favor a computer‐aided implant surgery approach.
Supporting patient/cross‐disciplinary expert survey results	N/A
Supporting/Contradicting	Computer assisted implant surgery provided significantly higher accuracy than free hand placement (Mahardawi et al. 2025). Static fully guided implant navigation surgery had the highest accuracy, followed by static half‐guided surgery. Freehand implant placement had the least accuracy (Gargallo‐Albiol et al. 2020).
Lack of literature	A completely free hand implant placement approach has not been compared to guided approaches in terms of PROs.
Evidence	Based on two systematic reviews (Gargallo‐Albiol et al. 2020; Mahardawi et al. 2025), fully guided approaches offered superior accuracy compared to pilot and traditional prosthesis guided approaches.
Recommended ClinROs	– Surgical/intraoperative complications – Clinician's treatment success
Recommended PROs	– Patient overall satisfaction with treatment – Patient‐reported complaints
Strength of consensus	Agree: 81% (Consensus) Agree: 73/Disagree: 15/Abstain: 1/Abstain (CoI): 1

Recommendation No. 4 (Aligned with current evidence).

**Table:** 

Domain	Treatment planning
Recommendation	For patients in need of maxillary arch rehabilitation with a complete implant‐supported fixed prosthesis, the number of implants should be based on several factors which include patient‐specific needs and clinical judgment. Whilst the number of appropriately distributed implants should be at least 4, we suggest 6 to reduce the potential for complications.
Supporting expert survey results	73% of experts supported using 6 implants for a fixed reconstruction.
Supporting patient/cross‐disciplinary expert survey results	N/A
Supporting/Contradicting	No implant‐supported rehabilitation of the edentulous maxilla (fixed or removable) should be supported on fewer than 4 implants. A one‐piece full‐arch fixed dental prosthesis can be supported by a minimum of 2 anterior axial plus 2 distally tilted implants, or by 6–8 axial implants symmetrically distributed through the posterior and anterior regions of the arch (Messias et al. 2021). Having a greater number of implants, as seen in the 6‐implant group, can lead to a decrease in technical and biological complications and reduce marginal bone loss (Sharaf et al. 2024). There were no differences in marginal bone loss and implant survival rates at 5 years, but 4 implants exhibited a significantly higher incidence of technical complications compared to 6 implants (Toia et al. 2025). When rehabilitating the edentulous maxilla with a full‐arch one‐piece prosthesis, most of the evidence supported the use of 4 implants (with two tilted distal implants) or 6 axial implants for a full‐arch one‐piece prosthesis (Ng et al. Forthcoming).
Lack of literature	N/A
Evidence	Based on three systematic reviews (Messias et al. 2021; Ng et al. Forthcoming; Sharaf et al. 2024) and one RCT (Toia et al. 2025), rehabilitation of the edentulous maxilla using 6 implants may be associated with less technical complications than 4 implants. Tilting of the most distal implants may be considered, angulated parallel to the frontal wall of the sinus, to reduce the invasiveness of bone augmentation procedures.
Recommended ClinROs	– Implant failure – Implant success – Implant survival – Mechanical/technical complications – Prosthetic failure – Prosthetic success – Prosthetic complications – Postoperative complications – Radiographic marginal bone loss – Surgical/intraoperative complications – Biological complications – Peri‐implant health (implant level) – Peri‐implant health (patient level) – Peri‐implant mucositis – Peri‐implant suppuration – Peri‐implantitis – Clinician's treatment success – Prosthodontic maintenance events/complications
Recommended PROs	– Chewing function/comfort/discomfort – Complications during treatment/maintenance – Ease of cleaning/oral hygiene efficiency – Patient overall satisfaction with treatment – Patient‐reported complaints – Prosthesis retention/stability – Quality of life (Oral Health‐Related Quality of Life, OHRQoL) – Speech/phonetics/pronunciation/function
Strength of consensus	Agree: 79% (Consensus) Agree: 68/Disagree: 17/Abstain: 0/Abstain (CoI): 1

Recommendation No. 5 (Aligned with current evidence).

**Table:** 

Domain	Treatment planning
Recommendation	For patients in need of maxillary arch rehabilitation with an implant overdenture, we suggest using at least 4 appropriately distributed implants to reduce implant failure.
Supporting expert survey results	Most experts (81%) favored 4 implants for an implant overdenture.
Supporting patient/cross‐disciplinary expert survey results	N/A
Supporting/Contradicting	**Supporting** Survival rate of implants appeared higher in the ≥ 4 implants group, but overdenture survival rate and patient satisfaction were unaffected (Di Francesco et al. 2019). Bar‐supported overdentures on 4 implants were not inferior to overdentures supported by 6 implants for rehabilitating the edentulous maxilla (Di Francesco et al. 2021). When rehabilitating the edentulous maxilla with an implant‐assisted denture, most of the evidence supported at least four implants with a bar attachment (Ng et al. Forthcoming). Implant loss rates for maxillary overdentures on < 4 implants were significantly higher than for 4 implants (Kern et al. 2016). No implant‐supported rehabilitation of the edentulous maxilla (fixed or removable) should be supported on fewer than 4 implants. Four to six (4–6) implants was the advised number to support an overdenture, whereas the use of mini implants in the maxilla was inadvisable (Messias et al. 2021). **Contradicting** Patients were satisfied with maxillary IODs on two implants (Zembic et al. 2019).
Lack of literature	N/A
Evidence	Based on five systematic reviews (Di Francesco et al. 2021, 2019; Kern et al. 2016; Messias et al. 2021; Ng et al. Forthcoming), maxillary implant overdentures should be supported by a minimum of 4 implants.
Recommended ClinROs	– Implant failure – Implant success – Implant survival – Mechanical/technical complications – Prosthetic failure – Prosthetic success – Prosthetic complications – Postoperative complications – Radiographic marginal bone loss – Surgical/intraoperative complications – Biological complications – Peri‐implant health (implant level) – Peri‐implant health (patient level) – Peri‐implant mucositis – Peri‐implant suppuration – Peri‐implantitis – Clinician's treatment success – Prosthodontic maintenance events/complications
Recommended PROs	– Chewing function/comfort/discomfort – Complications during treatment/maintenance – Ease of cleaning/oral hygiene efficiency – Patient overall satisfaction with treatment – Patient‐reported complaints – Prosthesis retention/stability – Quality of life (Oral Health‐Related Quality of Life, OHRQoL) – Speech/phonetics/pronunciation/function
Strength of consensus	Agree: 81% (Consensus) Agree: 69/Disagree: 14/Abstain: 1/Abstain (CoI): 1

Recommendation No. 6 (Could not be evaluated due to a lack of existing evidence).

**Table:** 

Domain	Treatment planning
Recommendation	For patients in need of maxillary arch rehabilitation requiring a complete implant‐supported prosthesis, we suggest presenting immediate implant placement as a treatment option to reduce overall treatment time when the clinical situation is favorable. **Comment** In patients considered for extraction of the maxillary dentition, we recommend proceeding after comprehensive evaluation of all clinical and patient‐specific factors.
Supporting expert survey results	N/A
Supporting patient/cross‐disciplinary expert survey results	N/A
Supporting/Contradicting	There were no significant differences in implant success and peri‐implant marginal bone loss between immediate and delayed implants with fixed full‐arch prostheses (Pellicer‐Chover et al. 2014).
Lack of literature	No studies were identified regarding the influence of timing of implant placement in the edentulous maxilla on PROs.
Evidence	Based on one RCT (Pellicer‐Chover et al. 2014) with a small sample size, immediate and delayed implant placement had no significant differences in terms of implant success and marginal bone loss.
Recommended ClinROs	– Implant failure – Implant success – Implant survival – Implant primary stability – Postoperative complications – Radiographic marginal bone loss – Surgical/intraoperative complications – Clinician's treatment success
Recommended PROs	– Esthetic satisfaction – Chewing function/comfort/discomfort – Complications during treatment/maintenance – Ease of cleaning/oral hygiene efficiency – Patient overall satisfaction with treatment – Patient‐reported complaints – Prosthesis retention/stability – Quality of life (Oral Health‐Related Quality of Life, OHRQoL) – Speech/phonetics/pronunciation/function
Strength of consensus	Agree: 77% (Consensus) Agree: 64/Disagree: 17/Abstain: 1/Abstain (CoI): 1

Recommendation No. 7 (Aligned with current evidence).

**Table:** 

Domain	Treatment Planning
Recommendation	For patients in need of maxillary arch complete implant‐supported prosthesis, to improve patient satisfaction/function, we suggest immediate implant placement and loading with a one‐piece screw‐retained provisional fixed prosthesis, provided that the clinical situation is favorable and primary implant stability is achieved. **Comment** In patients considered for extraction of the maxillary dentition, we recommend proceeding after comprehensive evaluation of all clinical and patient‐specific factors.
Supporting expert survey results	76% of experts favor immediate loading with a provisional restoration if primary stability is sufficient following immediate implant placement.
Supporting patient/cross‐disciplinary expert survey results	N/A
Supporting/Contradicting	Immediate implant loading with a fixed prosthesis in the edentulous maxilla was a reliable treatment option, with no significant differences in implant survival rates or marginal bone loss when compared to a conventional loading protocol (Jiang et al. 2021). There was some evidence that studies including edentulous patients rehabilitated with implant‐supported full‐arch FDPs demonstrated more satisfied patients with immediate than for the early or delayed loaded implant reconstructions (Gotfredsen et al. 2021). There was some evidence that patients were more satisfied with immediate compared early and delayed implant loading, but the timing of assessment may influence the outcome (Donos et al. 2021). Immediate loading of interantral implants yielded satisfactory results in the transition of patients from a failing maxillary dentition to full‐arch implant rehabilitation (Busenlechner et al. 2016b).
Lack of literature	N/A
Evidence	Based on three systematic reviews (Busenlechner et al. 2016b; Gotfredsen et al. 2021; Jiang et al. 2021) and one consensus conference (Donos et al. 2021), immediate implant loading in the edentulous maxilla has comparable implant survival rates to delayed protocols and is associated with higher patient satisfaction.
Recommended ClinROs	– Implant failure – Implant success – Implant survival – Mechanical/technical complications – Prosthetic failure – Prosthetic success – Prosthetic complications – Implant primary stability – Postoperative complications – Radiographic marginal bone loss – Clinician's treatment success – Prosthodontic maintenance events/complications
Recommended PROs	– Esthetic satisfaction – Chewing function/comfort/discomfort – Complications during treatment/maintenance – Ease of cleaning/oral hygiene efficiency – Patient overall satisfaction with treatment – Patient‐reported complaints – Prosthesis retention/stability – Quality of life (Oral Health‐Related Quality of Life, OHRQoL) – Speech/phonetics/pronunciation/function
Strength of consensus	Agree: 86% (Consensus) Agree: 72/Disagree: 10/Abstain: 2/Abstain (CoI): 0

Recommendation No. 8 (Cannot be evaluated due to a lack of existing evidence).

**Table:** 

Domain	Treatment planning
Recommendation	For patients in need of maxillary arch rehabilitation requiring a complete implant‐supported prosthesis in healed sites, provided implant primary stability is achieved and patient‐related factors are favorable, we suggest immediate loading with a one‐piece provisional screw‐retained fixed restoration to avoid complications related to postoperative use of a removable prosthesis.
Supporting expert survey results	N/A
Supporting patient/cross‐disciplinary expert survey results	N/A
Supporting/Contradicting	There was limited evidence of differences between immediate load and other loading regimens, regarding patient satisfaction and maintenance events/adversities (Abdunabi et al. 2019). Immediate loading of interantral implants in the nonaugmented edentulous maxilla yields favorable results comparable to delayed loading (Busenlechner et al. 2016a)
Lack of literature	N/A
Evidence	Based on two systematic reviews (Abdunabi et al. 2019; Busenlechner et al. 2016a), there were no differences in implant survival following immediate or delayed loading protocols.
Recommended ClinROs	– Implant failure – Implant success – Implant survival – Mechanical/technical complications – Prosthetic failure – Prosthetic success – Prosthetic complications – Implant primary stability – Postoperative complications – Radiographic marginal bone loss – Surgical/intraoperative complications – Clinician's treatment success – Prosthodontic maintenance events/complications
Recommended PROs	– Esthetic satisfaction – Chewing function/comfort/discomfort – Complications during treatment/maintenance – Ease of cleaning/oral hygiene efficiency – Patient overall satisfaction with treatment – Patient‐reported complaints – Prosthesis retention/stability – Quality of life (Oral Health‐Related Quality of Life, OHRQoL) – Speech/phonetics/pronunciation/function
Strength of consensus	Agree: 90% (Consensus) Agree: 78/Disagree: 8/Abstain:0/Abstain (CoI): 1

Recommendation No. 9 (Could not be evaluated due to a lack of existing evidence).

**Table:** 

Domain	Treatment planning
Recommendation	For patients in need of maxillary arch rehabilitation requiring a complete implant‐supported prosthesis, when performing immediate loading, we suggest using a one‐piece fixed screw‐retained cross‐arch provisional prosthesis splinting at least 4 implants with primary stability.
Supporting expert survey results	67% of experts use fixed provisional restorations when performing immediate loading.
Supporting patient/cross‐disciplinary expert survey results	While 58.5% of patients and 47.6% of cross‐disciplinary experts preferred a fixed provisional, 17.1% of patients and 19.1% of cross‐disciplinary experts favored a removable option. Notably, 24.4% of patients and 33.3% of cross‐disciplinary experts had no specific preference.
Supporting/Contradicting	Nil
Lack of literature	There was a lack of evidence comparing fixed to removable provisional restorations in the literature.
Evidence	The suggestion to use fixed provisional restorations for immediate loading cannot be adequately assessed due to insufficient supporting evidence.
Recommended ClinROs	– Implant failure – Implant success – Implant survival – Mechanical/technical complications – Prosthesis failure – Prosthesis success – Prosthetic complications – Implant primary stability – Postoperative complications – Radiographic marginal bone loss – Clinician's treatment success
Recommended PROs	– Esthetic satisfaction – Chewing function/comfort/discomfort – Complications during treatment/maintenance – Ease of cleaning/oral hygiene efficiency – Patient overall satisfaction with treatment – Patient‐reported complaints – Prosthesis retention/stability – Quality of life (Oral Health‐Related Quality of Life, OHRQoL) – Speech/phonetics/pronunciation/function
Strength of consensus	Agree: 95% (Consensus) Agree: 84/Disagree: 3/Abstain: 1/Abstain (CoI): 0

Recommendation No. 10 (Could not be evaluated due to a lack of evidence).

**Table:** 

Domain	Treatment planning
Recommendation	For patients in need of maxillary arch rehabilitation requiring a fixed complete implant‐supported prosthesis, we suggest an FP1/FP2 prosthesis design when the anatomical conditions allow, to avoid the need for extensive bone reduction/alveoloplasty.
Supporting Expert Survey Results	71% of experts prefer FP1 with minimal alveoloplasty over an FP3 design requiring alveoloplasty.
Supporting patient/cross‐disciplinary expert survey results	Both patients (95%) and cross‐disciplinary experts (86%) placed a high priority on the esthetic outcome of the prosthesis.
Supporting/Contradicting	Nil
Lack of literature	The literature lacks sufficient evidence directly comparing FP1 and FP3 prosthetic designs for the rehabilitation of the edentulous maxilla.
Evidence	Current literature lacks robust comparative studies definitively favoring FP1 over FP3. The preference for FP1 was largely guided by clinical experience, esthetic considerations, and minimal invasiveness rather than conclusive comparative evidence.
Recommended ClinROs	– Mechanical/technical complications – Plaque index/oral hygiene – Prosthesis failure – Prosthesis success – Prosthetic complications – Radiographic marginal bone level – Biological complications – Peri‐implant health (implant level) – Peri‐implant health (patient level) – Peri‐implant mucositis – Peri‐implant suppuration – Peri‐implantitis
Recommended PROs	– Esthetic satisfaction – Chewing function/comfort/discomfort – Complications during treatment/maintenance – Ease of cleaning/oral hygiene efficiency – Pain – Patient overall satisfaction with treatment – Patient‐reported complaints – Prosthesis retention/stability – Quality of life (Oral Health‐Related Quality of Life, OHRQoL) – Speech/phonetics/pronunciation/function
Strength of consensus	Agree: 90% (Consensus) Agree: 74/Disagree: 7/Abstain: 1/Abstain (CoI): 0

Recommendation No. 11 (Could not be evaluated due to a lack of evidence).

**Table:** 

Domain	Treatment procedure
Recommendation	For patients in need of maxillary arch rehabilitation with fixed complete arch or removable implant‐supported prosthesis, we recommend that the clinician assess the skeletal relationship, opposing dentition, implant distribution and prosthesis design in the selection of an appropriate occlusal scheme to reduce prosthetic complications.
Supporting expert survey results	70% of experts favor a bilaterally balanced group function when opposing an implant overdenture; however, no agreement was reached regarding the preferred occlusal scheme when opposing a fixed full‐arch mandibular prosthesis.
Supporting patient/cross‐disciplinary expert survey results	N/A
Supporting/Contradicting	Nil
Lack of literature	The literature lacks sufficient evidence regarding optimal occlusal scheme designs for the rehabilitation of the edentulous maxilla.
Evidence	
Recommended ClinROs	– Mechanical/technical complications – Prosthesis failure – Prosthesis success – Prosthetic complications
Recommended PROs	– Chewing function/comfort/discomfort – Patient overall satisfaction with treatment – Patient‐reported complaints – Quality of life (Oral Health‐Related Quality of Life, OHRQoL)
Strength of consensus	Agree: 100% (Strong consensus) Agree: 61/Disagree: 0/Abstain: 0/Abstain (CoI): 0

Recommendation No. 12 (Could not be evaluated due to a lack of evidence).

**Table:** 

Domain	Maintenance Care (Implant Overdentures)
Recommendation	For the completely edentulous patient with an implant retained removable maxillary overdenture, we recommend that the patient be informed that regular maintenance of the prosthesis is required. We suggest retention component replacement when the patient notices a loss of retention or when deemed necessary by the clinician.
Supporting expert survey results	89% of experts estimate that retention components require replacement within 5 years. However, the even spread and range of results between 1 and 5 years suggests significant patient variability.
Supporting patient/cross‐disciplinary expert survey results	N/A
Supporting/Contradicting	Nil
Lack of literature	No recent systematic reviews have been identified addressing the optimal timing for replacing retention components in maxillary implant overdentures.
Evidence	Current literature lacks evidence to guide the optimal timing for replacing retention components of maxillary implant overdentures.
Recommended ClinROs	– Prosthesis failure – Prosthesis success – Prosthetic complications
Recommended PROs	– Complications during treatment/maintenance – Patient overall satisfaction with treatment – Patient‐reported complaints – Prosthesis retention/stability
Strength of consensus	Agree: 89% (Consensus) Agree: 73/Disagree: 5/Abstain: 4/Abstain (CoI): 0

## 6 Summary of Areas for Future Research

Future research in full‐arch implant rehabilitation of the edentulous maxilla should focus on several key domains (Table 4). For treatment execution, further investigation is beneficial to determine the optimal number of implants required for fixed full‐arch prostheses, particularly in comparing the outcomes of four versus six or more implants in terms of surgical complexity, prosthetic success, patient satisfaction, and cost‐effectiveness. Additionally, the timing of implant placement—especially immediate placement in anterior versus posterior sites—requires robust evidence to clarify its impact on implant survival, bone preservation, and patient‐reported outcomes. Finally, in the maintenance phase, the ideal recall interval depending on specific patient risk, the influence of different prosthesis retrieval and peri‐implant care intervals on long‐term peri‐implant health, bone stability, and overall treatment success remains an important area for exploration.

**TABLE 4:** Summary of future research questions using the PICOS framework.

Domain	Research focus	P (Population)	I (Intervention)	C (Comparator)	O (Outcomes)	S (Study Design)
Treatment Planning and Execution	Optimum Number of Implants for Full‐Arch Prosthesis	Edentulous patients with full‐arch implant‐supported prostheses in the maxilla	Six implants or more	Four implants	Implant success, surgery duration, need for augmentation, prosthetic fit, complications, PROMs, cost‐effectiveness	RCTs, prospective case series, cohort studies
Treatment Planning and Execution	Timing of Implant Placement (Anterior vs. Posterior)	Edentulous patients with full‐arch implant‐supported prostheses in the maxilla	Immediate implant placement (Anterior vs. Posterior)	Early or late implant placement	Implant success/survival, augmentation needs, marginal bone loss, complications, surgery time, PROMs	RCTs, prospective case series, cohort studies
Maintenance	Prosthesis Retrieval at Different Recall Intervals	Edentulous patients with full‐arch implant‐supported prostheses in the maxilla	Retrieval and maintenance every X months	Alternative recall interval	Peri‐implant health, marginal bone loss, complications, implant success/survival, patient‐reported outcomes	Randomized controlled trial

## Author Contributions

**Nikos Donos:** conceptualization, methodology, supervision, project administration, writing – review and editing, writing – original draft, validation. **Ethan Ng:** writing – original draft, writing – review and editing, project administration, methodology, validation. **Claudio Mendes Pannuti:** methodology, writing – original draft, writing – review and editing, validation. **Giuseppe Alexandre Romito:** methodology, writing – review and editing, writing – original draft, validation. **Helena Cristina Oliveira Francisco:** methodology, writing – review and editing, writing – original draft, validation. **Samir Abou‐Ayash:** methodology, writing – review and editing, writing – original draft, validation. **Gustavo Avila‐Ortiz:** methodology, validation, writing – review and editing, writing – original draft. **Joao Manuel Mendez Carames:** methodology, validation, writing – original draft, writing – review and editing. **Paolo Casentini:** methodology, validation, writing – original draft, writing – review and editing. **Tali Chackartchi:** writing – original draft, methodology, validation, writing – review and editing. **Vivianne Chappuis:** methodology, validation, writing – review and editing, writing – original draft. **Stephen Chen:** writing – original draft, methodology, writing – review and editing, validation. **Paul Fugazzotto:** writing – original draft, methodology, validation, writing – review and editing. **William V. Giannobile:** methodology, validation, writing – review and editing, writing – original draft. **Yoshiyuki Hagiwara:** writing – original draft, writing – review and editing, validation, methodology. **Adam Hamilton:** writing – original draft, writing – review and editing, validation, methodology. **Saso Ivanovski:** writing – original draft, writing – review and editing, validation, methodology. **Sergio Kahn:** writing – original draft, writing – review and editing, validation, methodology. **Joseph Kan:** writing – original draft, writing – review and editing, validation, methodology. **France Lambert:** writing – original draft, writing – review and editing, validation, methodology. **Robert Alan Levine:** writing – original draft, writing – review and editing, validation, methodology. **Jose Manuel Navarro:** writing – original draft, writing – review and editing, validation, methodology. **Turker Ornekol:** writing – original draft, writing – review and editing, validation, methodology. **Michael Payer:** writing – original draft, writing – review and editing, validation, methodology. **Todd Schoenbaum:** writing – original draft, writing – review and editing, validation, methodology. **Manish Kumar Singh:** writing – original draft, writing – review and editing, validation, methodology. **Sejal Thacker:** writing – original draft, writing – review and editing, validation, methodology. **Gil Alcoforado:** writing – original draft, writing – review and editing, validation, methodology, conceptualization, supervision.

## Conflicts of Interest

All delegates disclosed secondary interests using the standardized ICMJE disclosure form. Potential conflicts of interest (CoI) were actively managed in accordance with Guidelines International Network (GIN) principles.

## Data Availability

The data that support the findings of this study are available from the corresponding author upon reasonable request.
